# Outcomes of Recurrent Laryngeal Nerve Reinnervation for Pediatric Unilateral Vocal Fold Paralysis

**DOI:** 10.1002/lary.70750

**Published:** 2026-07-13

**Authors:** Theodore A. Gobillot, Linda M. Carroll, Karen B. Zur

**Affiliations:** ^1^ Division of Otolaryngology Children's Hospital of Philadelphia Philadelphia Pennsylvania USA; ^2^ Department of Otorhinolaryngology—Head & Neck Surgery University of Pennsylvania Philadelphia Pennsylvania USA

**Keywords:** dysphonia, reinnervation, vocal fold paralysis

## Abstract

**Objectives:**

Recurrent laryngeal nerve (RLN) reinnervation has demonstrated high success rates for treatment of pediatric unilateral vocal fold paralysis (UVFP) in small cohorts. The study objective was to describe voice outcomes from the largest single‐center, single‐surgeon experience with RLN reinnervation for pediatric UVFP.

**Methods:**

This retrospective case series was conducted at a tertiary care children's hospital from 2007 to 2025. All patients who underwent RLN reinnervation for pediatric UVFP were included. Primary outcomes were perceptual and acoustic voice measures. Secondary outcomes included aspiration and dyspnea/stridor outcomes and identification of clinical factors associated with voice outcomes.

**Results:**

In total, 102 patients underwent RLN reinnervation. Seventy‐one patients (70%) had evaluable voice outcomes. “Moderate” to “dramatic” voice improvements were observed in 64/71 patients (90%). In the 53 patients with “dramatic” voice improvement, significant improvements were observed in pVHI (−15.9), GRBAS (−3.0), and Cepstral values (+2.1 dB), with 31/46 (67%) achieving near‐normal or normal Cepstral values. Female sex, decreasing gestational age, history of prolonged intubation, and presence of posterior intubation deficiency were associated with less robust voice outcomes. Aspiration was present in 25% of patients and resolved in 80% with follow‐up (12/15). Dyspnea/stridor suggestive of synkinesis was present in 5% of patients and resolved in all.

**Conclusion:**

RLN reinnervation is an effective intervention for treatment of pediatric UVFP. Patients with significant intubation history, posterior intubation deficiencies, and more severe prematurity are at higher risk for less than “dramatic” voice outcomes.

**Level of Evidence:**

4.

## Graphical abstract

The objective of this study was to describe voice outcomes from the largest single‐center, single‐surgeon experience with recurrent laryngeal nerve reinnervation for pediatric unilateral vocal fold paralysis. In 102 patients who underwent reinnervation, we demonstrate that reinnervation is an effective and durable intervention for treatment of pediatric unilateral vocal fold paralysis. Patients with significant intubation history, posterior intubation deficiencies, and more severe prematurity are at higher risk for less robust voice outcomes following reinnervation.

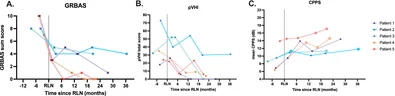

## 1 Introduction

Pediatric unilateral vocal fold paralysis (UVFP) causes significant dysphonia and dysphagia in children. UVFP often occurs in medically complex children, particularly those with cardiac disease, as most cases of vocal fold immobility are caused by iatrogenic surgical injury during cardiac or other mediastinal procedures [1, 2, 3]. The clinical presentation of UVFP is variable depending on the child's age and their ability to compensate for the paralysis. In children and adolescents, UVFP is associated with an increased risk of anxiety, inattention, social isolation, and negative self‐perception [4, 5].

Evaluation and management of pediatric UVFP require a multi‐disciplinary team including a pediatric laryngologist and speech‐language pathologist (SLP). While there remains no standardized management algorithm, there has been significant evolution in treatment paradigms for children with UVFP over the past two decades. RLN reinnervation was initially reported in the 1920s in adults and fell out of favor in the following decades due to disappointing results [6], but it has shown significant promise in pediatric patients with UVFP. While multiple studies in children have suggested that RLN reinnervation provides durable, high‐quality voice outcomes regardless of time since injury, these reports are largely based on perceptual data from small case series of 5–20 patients with short‐term follow‐up [7, 8, 9, 10, 11, 12, 13, 14, 15]. In fact, only 2 studies to date have examined acoustic outcomes in cohorts larger than 5 patients following RLN reinnervation [14, 15], and only a single study in 21 patients has examined outcomes using the now‐standard Cepstral Peak Prominence (CPP) [15]. A subset of studies have also evaluated swallowing outcomes following RLN reinnervation in patients with preoperative aspiration in the setting of UVFP and highlight the promise of reinnervation to resolve aspiration [16, 17].

The primary objective of this study is to describe perceptual and acoustic outcomes from the largest single‐center, single‐surgeon experience with RLN reinnervation for pediatric UVFP.

## 2 Materials and Methods

### 2.1 Study Participants

A retrospective case series was conducted on pediatric patients who underwent RLN reinnervation for UVFP at Children's Hospital of Philadelphia from January 2007 to October 2025. This study was approved by the Children's Hospital of Philadelphia Institutional Review Board. One patient was excluded who was noted to have UVFP with contralateral paresis. All clinical assessments, interventions, and follow‐up were performed by the senior author (K.B.Z.) as part of a dedicated multidisciplinary voice clinic. Prolonged intubation was defined as > 10 days. Prematurity severity was defined as extremely preterm (≤ 25 weeks), very preterm (26–31 weeks), moderately preterm (32–33 weeks), late preterm (34–36 weeks), and full term (37+ weeks). Voice outcomes were not evaluated for this study in patients with less than 4 months of follow‐up, who were less than 1 year post‐reinnervation, or were non‐verbal on follow‐up.

### 2.2 Clinical Assessment

All patients were evaluated in a multidisciplinary voice clinic, where each patient underwent a laryngeal assessment and full perceptual and acoustic voice assessments jointly with certified, licensed SLPs who have over 10 years of specialization in pediatric voice disorders. Pediatric patients with unresolved and uncompensated UVFP leading to significant dysphonia, aspiration, and/or dyspnea/stridor with a clinical quality of life impact were offered a confirmatory laryngeal electromyography (EMG), RLN reinnervation, and possible concurrent gel injection laryngoplasty. For those patients with evidence of preoperative aspiration, a clinical assessment jointly with an SLP was performed to determine a safe diet for the patient.

### 2.3 Pediatric Voice Handicap Index (pVHI)

The pVHI was used pre‐ and postoperatively to determine perceived voice disability. The pVHI is a validated parental proxy for dysphonia severity with subdomains focused on the functional, physical, and emotional aspects of the child's dysphonia [18]. These assessments were completed by a parental proxy prior to the initial evaluation and prior to each postoperative follow‐up appointment or at regular intervals for patients/families following up remotely.

### 2.4 Grade–Roughness–Breathiness–Aesthenia–Strain (GRBAS) Scoring

The perceptual rating scale of GRBAS score was completed by the voice care team pre‐ and postoperatively to determine a qualitative assessment of overall dysphonia [19]. For patients unable to attend in‐person follow‐up appointments, GRBAS scoring was performed on voice recordings sent via secure messaging.

### 2.5 Acoustic Assessments

Smoothed Cepstral Peak Prominence (CPPS) was the primary acoustic measure used in this study. CPPS is a more reliable measure of dysphonia than previous perturbation measures that rely on measuring only sustained vowels and require a known fundamental frequency [20, 21]. CPPS values were obtained from either replicates of comfortable sustained phonation on /a/ (if available) or CAPE‐V sentence (“We were away a year ago,” etc.). Praat software (Version 6.4.26) was used to calculate CPPS. Maximum phonation time was also calculated using a comfortable pitch and loudness for maximum sustained /a/. CPPS was compared to published sex and age‐matched normative values in non‐dysphonic pediatric patients [20, 22, 23]. This was examined pre‐ and postoperatively at scheduled follow‐up visits. For patients unable to attend in‐person follow‐up appointments, CPPS was calculated based on voice recordings sent via secure messaging.

Overall, for patients who could not attend postoperative follow‐up visits, subjective and objective voice outcomes (via recordings that were sent securely to the team at regular intervals postoperatively) were reviewed and progress was discussed remotely with the family to determine if further therapy was indicated.

### 2.6 Recurrent Laryngeal Nerve Surgical Management

The recurrent laryngeal nerve reinnervation procedure consisted of a transoral laryngeal EMG done via suspension to confirm chronic denervation injury to the paralyzed vocal fold, possible injection laryngoplasty with carboxymethylcellulose voice gel (Prolaryn, Merz Therpeutics) to the paralyzed fold, and reinnervation by anastomosis of a healthy ansa cervicalis donor nerve to a proximal segment of the injured RLN.

The surgical procedure has been previously described by our team [24]. A suspension microlaryngoscopy was performed in order to obtain a thorough evaluation of the larynx, specifically to assess arytenoid joint mobility, and to rule out a posterior intubation deficiency or vocal fold scarring. Next, a laryngeal EMG was performed to confirm the chronic denervation of the affected vocal fold. Finally, the recurrent laryngeal nerve reinnervation was performed with an ansa cervicalis branch as the donor in an end‐to‐end fashion. Patients were discharged on the day of surgery with no restriction to the diet but with resumption of the preoperative restriction if present (i.e., those who aspirated preoperatively continued thickening the diet until cleared by the speech pathologist as noted below; those who did not aspirate preoperatively had no dietary restrictions).

During the surgical consent process, the possible merits of gel injection, lasting 2–3 months, were discussed with the parents, with the notion with the gel would likely provide a temporary augmentation of vocal strength while the reinnervation starts to take effect. The off‐label use of an injection material in the pediatric population was reviewed as well. The timeline of expected benefits from reinnervation, starting at 3–6 months and continuing until 20 months postoperatively, was discussed with families. Additionally, the families were counseled that if a posterior intubation deficiency or vocal scarring was noted, there could be an impact on voice outcomes following reinnervation.

### 2.7 Postoperative Care

The typical follow up was set at 1–2 months post reinnervation, and then every 6 months for a total of 2 years postoperatively. At each follow‐up visit, pVHI, GRBAS, and CPPS were measured as described above. For patients who traveled nationally or internationally for care at our institution, the postoperative follow up was often accomplished remotely via virtually and securely submitted pVHI and voice recordings for GRBAS and CPPS analysis at the same intervals as the in‐person visits. For patients diagnosed with silent aspiration preoperatively, a modified barium swallow (video swallow study) was recommended around 5–8 months post reinnervation, corresponding to the improved phonation related to the reinnervation itself and beyond the timeframe for resorption of the gel injection. For those patients who had clinical aspiration, a bedside assessment with a SLP was made at a similar time, but without imaging, and a plan was made for de‐escalation of thickening based on results. Visualization of the larynx was not performed routinely in the early postoperative course unless there were specific concerns. This was mostly to avoid patient anxiety, and the information from a laryngoscopy/stroboscopy did not change the management in the first 2 years postoperatively.

### 2.8 Integrated Clinical Assessment Groupings

Patients were grouped according to an integrated clinical assessment at their most recent follow‐up evaluation in voice clinic. This is because no single perceptual or acoustic measurement of voice is sufficient to describe a patient's voice outcome in isolation. Patients were classified as having “dramatic” improvement if they had no, minimal, or mild dysphonia with clear significant improvement at last in‐person or remote follow‐up. Patients with “moderate” improvement had persistence of dysphonia described as moderate at last follow‐up that was still a clear incremental improvement from their baseline. Finally, patients with “minimal/no” improvement were clearly described as having no or minimal change to their voice at last follow‐up.

### 2.9 Statistical Analysis

Statistical comparisons were performed in Prism (GraphPad). Welch's *t*‐test was used to compare voice outcomes between integrated clinical assessment groups. Simple logistic regression was used to examine associations between clinical factors and less robust voice outcomes. One‐way analysis of variance (ANOVA) followed by Tukey's post hoc test for multiple comparisons was used to compare outcomes in patients with “dramatic” voice improvement with varying follow‐up.

## 3 Results

### 3.1 Overall Cohort Demographics and Characteristics

A total of 102 patients underwent RLN reinnervation at our institution from 2007 through October 2025. Table 1 summarizes demographics and relevant clinical characteristics. Average age of reinnervation was 9.5 years old (median 8.2 years old, range 1.9–20.8 years). Seven patients were over the age of 18 (18.1–20.8 years) and were included because the onset of the RLN injury was as a child. The majority of UVFP was caused by a prior cardiac procedure (89%), most commonly patent ductus arteriosus (PDA) ligation. Most patients (70%) carried a history of prematurity (within patients with history of prematurity: median gestational age of 25 weeks, range 22–36 weeks; 43% with history of extreme prematurity) and 59% had a history of prolonged intubation. There was a high rate of co‐occurring aspiration as an indication for reinnervation (25%), and a small number of patients (6%) with dyspnea/stridor (suggestive of synkinesis) preoperatively. At the time of the analysis, there were 71 patients that had > 4 months of follow‐up and voice outcomes following reinnervation were examined in these patients. Within this group of 71 patients, the mean follow up time was 31 months with the longest follow‐up time at 17 years postoperatively.

**TABLE 1:** Demographics and clinical characteristics.

	No. of patients (*n* = 102)
Mean age at time of surgery, years (range)	9.5 (1.9–20.8)
Sex	
Male (%)	55 (54%)
Female (%)	47 (46%)
Cause of paralysis	
Iatrogenic—cardiac (%)	91 (89%)
Iatrogenic—other (%)	6 (6%)
Idiopathic (%)	5 (5%)
Median gestational age within patients with prematurity, weeks (range) (*n* = 71)	25 (22–36)
Extremely preterm (≤ 25 weeks)	44 (43%)
Very preterm (26–31 weeks)	14 (14%)
Moderately preterm (32–33 weeks)	3 (3%)
Late preterm (34–36 weeks)	10 (10%)
Full term (37+ weeks)	28 (27%)
Unknown	3 (3%)
History of prolonged intubation	60 (59%)
Mean length of prolonged intubation (months)	2.7
History of tracheostomy (%)	4 (4%)
Evidence of aspiration (%)	25 (25%)
Evidence of dyspnea and/or stridor (%)	6 (6%)

### 3.2 Integrated Clinical Assessment of Voice to Stratify Patient Outcomes

Seventy‐one patients had evaluable voice outcomes, as patients with less than 4 months of follow‐up (and without any follow‐up acoustic data), < 1‐year post‐reinnervation, or were non‐verbal at follow‐up were not further evaluated for this study (Figure 1). “Dramatic” voice improvement was observed in 53 (53/71, 75%) patients, “moderate” improvement in 11 (11/71, 15%), and “no/minimal” change in 7 (7/71, 10%). Two patients were non‐verbal at follow‐up and could not be assessed. Of note, some patients were aphonic at baseline and thus do not have both pre‐ and postoperative acoustic data, and other patients did not always submit both perceptual and acoustic data for analysis.

**FIGURE 1:**
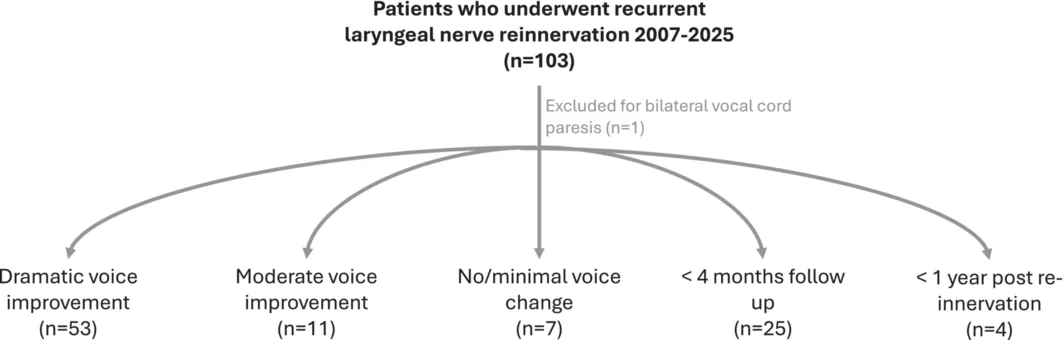
Overview of groupings of patients in overall voice outcomes based on integrated clinical assessment.

### 3.3 Perceptual and Acoustic Outcomes by Integrated Clinical Assessment Groupings

The perceptual and acoustic outcomes in each group were concordant with their integrated clinical assessment group (Table 2). From a perceptual outcomes standpoint, patients with “dramatic” improvement in voice had a mean net decrease of ~16 points on pVHI and of ~3 points on GRBAS. In the group of 46 patients with “dramatic” voice improvement who had acoustic recordings for analysis, CPPS was in the normal range for 22/46 (48%) and was nearly normal for 9/46 (20%). CPPS mean net change was +2.1 dB and maximum phonation time was +2.9 s for this group. All perceptual and acoustic changes in the “dramatic” improvement group were significant (except for GRBAS strong trend, *p* = 0.05) when compared to the remaining patients. The “moderate” and “no/minimal” improvement groups were small and underpowered to compare to each other for differences; however, notable differences were present in pVHI net change (+1.1 points in moderate improvement, +11.2 in no/minimal improvement), GRBAS net change (−2.0 points in moderate, −0.2 in no/minimal improvement), and CPPS net change (+0.5 dB in moderate, −2.1 in no/minimal improvement). Thus, the “moderate” group had perceptual and acoustic improvements. Within the no/minimal improvement group, three patients underwent repeat injections.

**TABLE 2:** Perceptual and acoustic outcomes of patients in each clinical category.

	Dramatic improvement (*n* = 53)^a^	Moderate improvement (*n* = 11)^b^	No/minimal improvement (*n* = 7)^c^	*p* (dramatic improvement vs. others)
pVHI survey total, mean net change	−15.9	+1.1	+11.2	< 0.0001
pVHI‐F	−6.1	−1.3	+2.7	
pVHI‐P	−5.5	+1.9	+3.2	
pVHI‐E	−4.2	+0.5	+5.3	
GRBAS sum score, mean net change	−3.0	−2.0	−0.2	0.05
CPPS within non‐dysphonic range for sex and age, no. (%)	22 (48%)	0	0	< 0.0001
CPPS < 1 dB from normal range, no. (%)	9 (20%)	1 (10%)	0	
CPPS mean net change (dB)	+2.1	+0.5	−2.1	0.006
Maximum phonation time mean net change (s)	+2.9	−0.9	+0.5	0.002

^a^ 41/53 patients had pVHI, 41/53 had GRBAS scores, 46/53 had mean CPPS values that were able to be evaluated according to a reference range, 39/53 had both pre‐ and postoperative mean CPPS values, and 29/53 had pre‐ and postoperative maximum phonation time values.

^b^ 8/11 patients had pVHI, 7/11 had GRBAS scores, 10/11 had mean CPPS values that were able to be evaluated according to a reference range, 6/11 had both pre‐ and postoperative mean CPPS values, and 5/11 had pre‐ and postoperative maximum phonation time values.

^c^ 6/7 patients had pVHI and GRBAS scores, 6/7 had mean CPPS values that were able to be evaluated according to a reference range, 6/7 had both pre‐ and postoperative mean CPPS values, and 4/7 had pre‐ and postoperative maximum phonation time values.

Without considering clinical assessment groupings, 32/62 (52%) of all patients with evaluable acoustics have a normal or near‐normal voice at most recent follow‐up, and the mean pVHI and GRBAS net changes at most recent follow‐up were −10.5 and −2.6, respectively.

### 3.4 Clinical Characteristics Associated With Less Robust Outcomes Following Reinnervation

To better understand factors that may influence voice outcomes following RLN reinnervation, we examined several characteristics using simple logistic regression (Table 3). Female sex (*p* = 0.03), decreasing gestational age (*p* = 0.01), prolonged intubation history (*p* = 0.008), and presence of posterior intubation deficiency at time of reinnervation (*p* = 0.04) were associated with increased odds of less robust voice outcomes following reinnervation (no/mild or moderate improvement). There was no association between less robust outcomes and age, length of time between RLN injury and reinnervation, and length of intubation. Of note, the average time interval between RLN injury and reinnervation was 8 years (range 0.7–20.5 years).

**TABLE 3:** Clinical factors associated with less robust voice outcomes.

	Odds ratio (95% confidence interval)	*p*
Age	0.98 (0.88–1.09)	0.74
Time from recurrent laryngeal nerve injury to reinnervation	1.04 (0.93–1.15)	0.52
Male sex	0.29 (0.08–0.98)	0.03
Gestational age	0.89 (0.80–0.98)	0.01
Prolonged intubation	5.19 (1.50–24.33)	0.008
Length of intubation	1.45 (0.90–2.43)	0.12
Presence of posterior intubation deficiency	4.63 (1.22–21.72)	0.04

### 3.5 Acoustic and Perceptual Outcomes in Patients With “Dramatic” Voice Improvement Over Increasing Follow‐Up Time

Following RLN reinnervation, there is a period of gradual improvement in voice quality that is usually first noted 3–6 months postoperatively and continues for as long as 20 months. To determine if this gradual improvement in outcomes was evident in our cohort who experienced overall “dramatic” voice improvements, we analyzed the latest follow‐up perceptual and acoustic outcomes at 6–12 months, 12–20 months, and > 20 months postoperatively (Table 4, Figure 2). We found similarly excellent reduction in pVHI across all follow‐up groups. GRBAS scores decreased and an increasing number of patients achieved normal Cepstral values with increasing follow‐up. The decrease in GRBAS scores > 20 versus 6–12 months follow‐up was the only significant finding on further statistical testing (Figure 2). Figure 3 shows representative perceptual and acoustic outcomes over time for five patients with “dramatic” improvements in voice. No patients with dramatic voice improvements had a decrease in voice outcomes over time. There was one male patient who underwent reinnervation before puberty and had a dramatic voice improvement. The patient re‐presented at time of pubertal voice changes and was recommended voice therapy with excellent outcome.

**TABLE 4:** Perceptual and acoustic outcomes of patients with dramatic improvement at last follow‐up.

	6‐12 months follow‐up^a^	12‐20 months follow‐up^b^	> 20 months follow‐up^c^
pVHI survey total, mean net change	−15.4	−20.6	−16.1
pVHI‐F	−5.9	−7.2	−6.2
pVHI‐P	−4.8	−6.8	−6.3
pVHI‐E	−4.7	−6.6	−3.6
GRBAS scale, mean net change	−1.4	−2.8	−4.8
CPPS within non‐dysphonic range, no. (%)	4 (27)	5 (71)	10 (63)
CPPS < 1 dB from normal range, no. (%)	2 (13)	1 (14)	5 (32)
CPPS mean net change (dB)	+1.4	+2.4	+3.1
Maximum phonate time mean net change (s)	+1.5	+2.7	+4.5

^a^ pVHI based on 16 patients, GRBAS based on 15 patients, CPPS ranges based on 15 patients, CPPS net change based on 13 patients, and maximum phonation time based on 9 patients.

^b^ pVHI based on five patients, GRBAS for eight patients, CPPS ranges for seven patients, CPPS net change for five patients, and maximum phonation time for three patients.

^c^ pVHI based on 18 patients, GRBAS based on 16 patients, CPPS ranges based on 16 patients, CPPS net change based on 14 patients, and maximum phonation time based on 12 patients.

**FIGURE 2:**
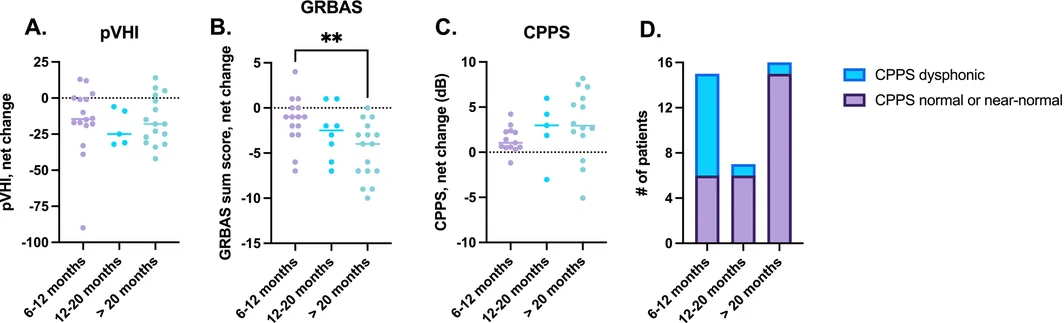
Comparison of pVHI (A), GRBAS (B), CPPS (C) and CPPS categorization as normal/near‐normal or dysphonic (D) at different follow‐up intervals in patients with “dramatic” voice improvement. **p < 0.01. [Color figure can be viewed in the online issue, which is available at www.laryngoscope.com]

**FIGURE 3:**
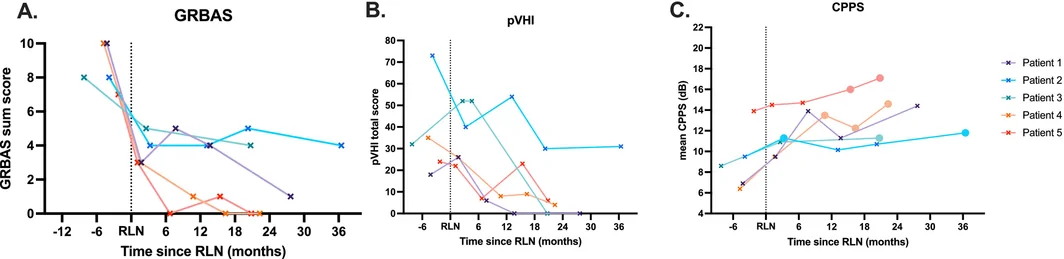
Representative examples of (A) GRBAS, (B) pVHI, and (C) mean CPPS over time of five patients with “dramatic” improvement in voice and > 20 months of follow‐up. Solid dot points in (C) represent normal CPPS values for patient age. [Color figure can be viewed in the online issue, which is available at www.laryngoscope.com]

### 3.6 Aspiration and Dyspnea/Stridor Outcomes

Approximately 25% of patients had evidence of aspiration (modified barium swallow, functional endoscopic swallow study) preoperatively. Of the 15 evaluable patients who were not lost to follow‐up, 12 (80%) had resolution of aspiration both clinically and on follow‐up radiographic evaluation when indicated. Two patients are awaiting further follow‐up to determine if their aspiration resolves following surgery. Dyspnea and/or stridor were present in 6 patients and resolved in all of them (Table 5).

**TABLE 5:** Aspiration and synkinesis outcomes.

	No. of patients (*n* = 102)
Evidence of aspiration, no. (%)	25
Resolved	12 (48%)
Awaiting further follow‐up	2 (8%)
Did not resolve	1 (4%)
Lost to follow‐up	10 (40%)
Evidence of synkinesis, no. (%)	6
Resolved	6 (100%)

## 4 Discussion

Management of pediatric UVFP has traditionally relied on either close observation, voice therapy, and/or surgical intervention. For children with uncompensated glottic insufficiency who may also have co‐occurring aspiration and dyspnea/stridor, surgical intervention is strongly recommended. Several studies over the past 15 years have provided the first evidence that RLN reinnervation can offer children durable, high‐quality voice and swallow outcomes [7, 8, 9, 12, 13, 14, 15, 16, 17, 25]. However, these studies are limited by either small patient cohorts, a lack of both perceptual and acoustic data, and/or limited long‐term patient follow‐up. In this study, we present the largest single‐institution, single‐surgeon experience spanning 20 years of RLN reinnervation for pediatric patients. This includes perceptual, acoustic, dysphagia, and dyspnea/stridor outcomes over a mean follow‐up time of 31 months, with the longest follow‐up time approaching 17 years postoperatively. We also identify patient factors that are associated with less than “dramatic” improvement following reinnervation, which are valuable in both patient selection and providing guidance to this complex patient population and their families.

From a voice outcomes standpoint, our data is in agreement with several previously published studies, most notably Ongkasuwan et al. [15], which is the only report of acoustic outcomes using the now‐standard CPP in patients who underwent RLN reinnervation. In the pediatric population, current normative studies report CPPS ranges from 13.8–17.8 dB on sustained /a/ from 3 to 17.9 years of age [23]. In Ongkasuwan et al.'s experience with 21 patients over a mean follow‐up period of 13 months, CPP increased by 2.9 dB and in MPT by 1.9 s. The CPP increases observed in this study and our report are significantly more than what would be expected by increasing age alone, as CPP has been shown to increase approximately 0.240 dB per year in males and 0.168 dB per year in female [23]. Several systematic reviews and meta‐analyses have attempted to integrate evidence from multiple smaller studies to examine patient outcomes following RLN reinnervation [8, 9]. While the data remains very heterogeneous, these reviews demonstrate a clear perceptual voice benefit. Hoey et al. found a mean GRBAS score improvement by 3.6 points across 27 patients from 5 studies [9]. Guarino et al. published the largest systematic review and meta‐analysis of RLN reinnervation for UVFP to date comprised of both adult and pediatric patients, and demonstrated significant improvements in pVHI and MPT post‐reinnervation for pediatric patients [8]. Of note, both studies only report MPT as an objective voice outcome which is confounded by age in the pediatric population [26, 27].

Our data provide further support to the expected timeline of vocal benefits following reinnervation. Many patients can expect to see some immediate benefits from gel injection laryngoplasty at the time of reinnervation. We excluded data from less than 4 months postoperatively to avoid confounding the data by the effects of the injection. This benefit is no longer present by ~3–4 months postoperatively, and voice and swallow benefits from the reinnervation can be seen as early as 3–6 months after surgery. These benefits are expected to continue to accrue gradually until as long as 20 months after surgery at which point there is a plateau in further gains. We were able to capture these benefits by examining acoustic outcomes in patients with “dramatic” voice improvements 6–12, 12–20, or > 20 months after surgery. Many of these associations were not statistically significant, likely due to small numbers of patients in each sub‐group. The first patients who had “dramatic” benefit from this procedure have been followed for as long as 17 years, highlighting the durability of the reinnervation procedure.

We have anecdotally observed that patients with a more significant history of prematurity tend to have a higher risk for persistent breathy dysphonia even after reinnervation. This is likely multi‐factorial, including contributions from lower respiratory pathology and breath support; however, a subset of these patients have posterior intubation deficiencies and vocal fold scarring from prolonged intubation perinatally, which may impact eventually achieving full glottic closure after reinnervation. We previously demonstrated that a higher grade posterior intubation deficiency is associated with decreased perceptual voice outcomes following phonosurgery for vocal fold paralysis [28]. Here, we extend these findings to show that patients with more significant prematurity, a history of prolonged intubation, and the presence of posterior intubation deficiency at the time of reinnervation are more likely to have less robust voice outcomes following reinnervation. This should be incorporated in pre‐ and postoperative counseling for patients but does not represent contraindications for reinnervation. If reinnervation is not effective, further laryngeal intervention, including framework surgery when the patient is nearing or at adult age, can be considered. Interestingly, the length of intubation was not significantly different in voice outcomes in our cohort, suggesting that perhaps the gestational age of intubation is a more critical determinant. Premature infants born younger than 28 weeks' gestation have reduced laryngeal development of macula flava which may predispose this population to intubation‐related deficiencies. Future studies with larger numbers of patients are needed to clarify this relationship. The time interval between RLN injury and reinnervation did not impact voice outcomes.

Approximately 25% of patients in this study had evidence of aspiration preoperatively. Aspiration resolved in 80% of patients who underwent reinnervation and were not lost to follow‐up. This adds to a growing body of literature that shows reinnervation is an effective intervention for aspiration [16, 17]. All patients with clinical evidence of dyspnea/stridor potentially consistent with synkinesis experienced resolution of these symptoms postoperatively.

### 4.1 Limitations

There are several limitations to this study that must be considered. This includes heterogeneity in follow‐up across the cohort, including a significant number of patients with less than 4 months of follow‐up data, and incompleteness of some datasets (given national and international draw). The study is retrospective in nature and thus is subject to limitations from this study type.

## 5 Conclusion

Recurrent laryngeal nerve reinnervation in the pediatric population is an effective intervention for treatment of dysphonia, aspiration, and dyspnea/stridor caused by UVFP. Patients with a significant intubation history, posterior intubation deficiency, and more severe prematurity are at higher risk for less robust voice outcomes following reinnervation.

## Funding

The authors have nothing to report.

## Conflicts of Interest

The authors declare no conflicts of interest.

## Data Availability

The data that support the findings of this study are available from the corresponding author upon reasonable request.
